# Harvesting methods of umbilical cord-derived mesenchymal stem cells from culture modulate cell properties and functions

**DOI:** 10.1016/j.reth.2024.05.010

**Published:** 2024-05-27

**Authors:** Mitsuyoshi Nakao, Kenichi Nagase

**Affiliations:** a Faculty of Pharmacy, Keio University, 1-5-30 Shibakoen, Minato-ku, Tokyo, 105-8512, Japan; b Graduate School of Biomedical and Health Sciences, Hiroshima University, 1-2-3 Kasumi, Minami-ku, Hiroshima, 734-8553, Japan

**Keywords:** Mesenchymal stem cell, Cytokine expression, Umbilical cord, Temperature responsive cell culture dish, Enzymatic digestion, Cell sheet

## Abstract

**Introduction:**

Human umbilical cord-derived mesenchymal stem cells (UC-MSCs) are promising candidates for stem cell therapy. Various methods such as enzymatic treatment, cell scraping, and temperature reduction using temperature-responsive cell culture dishes have been employed to culture and harvest UC-MSCs. However, the effects of different harvesting methods on cell properties and functions *in vitro* remain unclear. In this study, we investigated the properties and functions of UC-MSC using various cell-harvesting methods.

**Methods:**

UC-MSC suspensions were prepared using treatments with various enzymes, cell scraping, and temperature reduction in temperature-responsive cell culture dishes. UC-MSC sheets were prepared in a temperature-responsive cell culture dish. The properties and functions of the UC-MSC suspensions and sheets were assessed according to Annexin V staining, lactate dehydrogenase (LDH) assay, re-adhesion behavior, and cytokine secretion analysis via enzyme-linked immunosorbent assay.

**Results:**

Annexin V staining revealed that accutase induced elevated UC-MSC apoptosis. Physical scraping using a cell scraper induced a relatively high LDH release due to damaged cell membranes. Dispase exhibited relatively low adhesion from initial incubation until 3 h. UC-MSC sheets exhibited rapid re-adhesion at 15 min and cell migration at 6 h. UC-MSC sheets expressed higher levels of cytokines such as HGF, TGF-β1, IL-10, and IL-6 than did UC-MSCs in suspension.

**Conclusions:**

The choice of enzyme and physical scraping methods for harvesting UC-MSCs significantly influenced their activity and function. Thus, selecting appropriate cell-harvesting methods is important for successful stem cell therapy.

## Highlights

• Umbilical cord-derived mesenchymal stem cells (UC-MSCs) harvested by various methods were characterized.
• Enzymes and cell scraping induced UC-MSC damage compared to cells exposed to temperature changes.
• UC-MSC sheets secreted greater amounts of cytokines than did cells cultured in suspension.

## Graphical abstract

**Figure:**
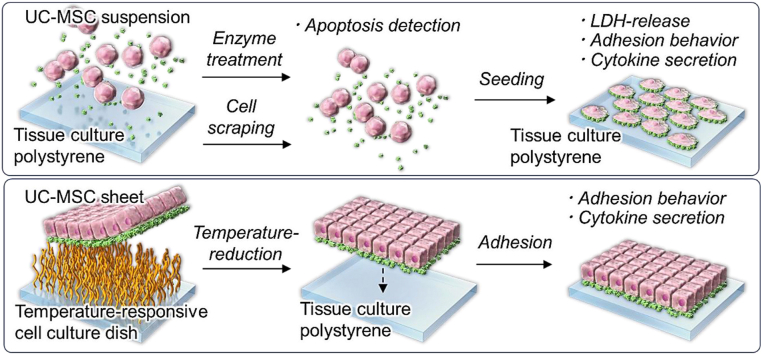

## 1 Introduction

Mesenchymal stem cells (MSCs) have been investigated for their therapeutic effectiveness due to their ability to secrete various therapeutic cytokines for cell proliferation, neoangiogenesis, inflammatory suppression, and immunoregulation [[1], [2], [3], [4], [5], [6], [7]]. MSCs can be harvested from various tissue types such as bone marrow, adipose tissue, umbilical cord, and blood [8]. UC-MSCs exhibit higher proliferative capacity and cell viability than do bone marrow-derived MSCs (BM-MSCs) and adipose tissue-derived MSCs (AD-MSCs) [[9], [10], [11], [12], [13], [14]]. Furthermore, UC-MSCs exhibited higher levels of secreted therapeutic cytokines and a higher proliferative capacity than that of BM-MSCs and AD-MSCs [10]. Additionally, UC-MSCs secrete higher levels of therapeutic cytokines than do the other two MSC types [10]. UC-MSCs are obtained non-invasively from postnatally acquired umbilical cords and are typically discarded as routine waste tissue after childbirth [15].

The therapeutic benefits of MSCs have been extensively investigated using various methods, including systemic and local cell administration via cell suspension injections. Cell sheets, monolayer cellular tissues, and transplantation are also effective administration methods for patients due to their high cytokine engraftment and secretion [[16], [17], [18], [19], [20], [21], [22], [23], [24], [25], [26], [27], [28], [29]]. Cell sheets were prepared using cell culture dishes modified with thermoresponsive poly(*N*-isopropylacrylamide) (PNIPAAm). PNIPAAm exhibits reversible hydration/dehydration changes in response to cell culture temperatures near 37 °C [30]. The unique properties of PNIPAAm have been exploited in the context of biomedical applications such as drug delivery systems [[31], [32], [33], [34], [35], [36], [37]], bioanalysis and biosensor devices [[38], [39], [40], [41], [42], [43], [44]], nano-actuators [[45], [46], [47], [48]], bioseparation tools [[49], [50], [51], [52], [53], [54]], cell-separation materials [[55], [56], [57], [58], [59]], cell culture substrates [[60], [61], [62], [63], [64], [65], [66], [67], [68], [69]], and cell sheet therapy in diverse types of regenerative medicine [[16], [17], [18], [19], [20], [21], [22],[24], [25], [26], [27], [28]]. For cell sheet preparation, the cells were seeded onto PNIPAAm-modified dishes. The cells were allowed to proliferate until they reached confluence. Upon reaching confluence, the cell sheet was harvested by lowering the temperature from 37 °C to 20 °C. This is based on the knowledge that the modified PNIPAAm on the culture dish surfaces became hydrophilic at 20 °C, thus leading to reduced cell adhesion to the dish. Cell sheets maintain their cellular activity and extracellular matrix, as cell sheet harvesting can be performed seamlessly by changing the temperature without destroying the cellular structures. In contrast, cell harvesting using digestive enzymes such as trypsin reduces the activity of cells and destroys cellular structures. This is due the observation that enzymes destroy proteins around cells, including the extracellular matrix [70]. Therefore, the cell harvesting methods after cell culture are likely to influence the activity of the harvested cells.

In this study, we investigated the properties and functions of UC-MSC using various harvesting methods, including enzymatic treatment, physical scraping with cell scrapers, and temperature reduction (TR) using PNIPAAm-modified dishes. Apoptosis, cell membrane injury, cell adhesion, and cytokine secretion by UC-MSCs were investigated in response to different cell harvesting methods. Effective methods for harvesting UC-MSC have been investigated to enhance their potential for stem cell therapy.

## 2 Materials and methods

### 2.1 Cell culture

Human MSCs derived from Wharton's jelly of the umbilical cord were purchased from PromoCell (Heidelberg, Germany). UC-MSCs were cultured in Dulbecco's Modified Eagle's Medium (DMEM; Gibco, Waltham, MA, USA) supplemented with 10% fetal bovine serum (FBS; Gibco), 1% GlutaMAX (Gibco), 1% MEM non-essential amino acids (Gibco), 100 units/mL penicillin, and 100 μg/mL streptomycin (Gibco). UC-MSCs were incubated at 37 °C under 5% CO_2_ in a humidified chamber and passaged when the cells reached confluence. UC-MSCs were then harvested by digestive enzyme (TrypLE, Gibco) treatment for 5 min and seeded into other tissue culture dishes at a density of 4000–6000 cells/cm^2^ between passages 3 and 6 this study.

### 2.2 Preparation of cell suspensions with temperature reduction, chemical, or physical harvesting

**Fig. 1:**
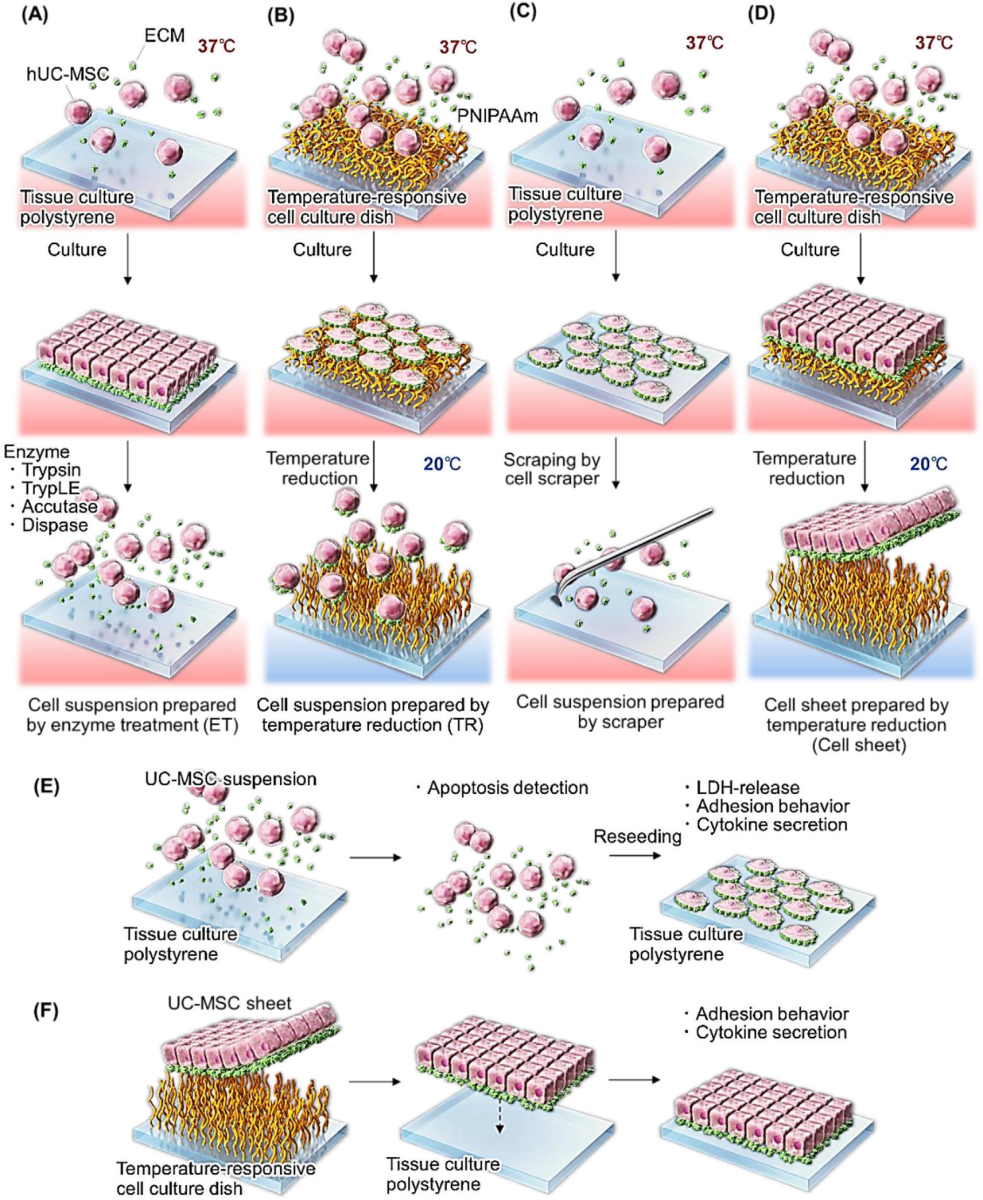
Schematic illustration of the preparation scheme for (A) UC-MSC suspension by enzyme treatment, (B) UC-MSC suspension by temperature treatment in a temperature-responsive cell culture dish, and (C) UC-MSC suspension by scraping with a cell scraper. (D) UC-MSC sheet cultured in a temperature-responsive cell culture dish. (E) Scheme of UC-MSC suspension characterization and (F) Scheme of UC-MSC sheet characterization by observing adhesion to culture plates and cytokine secretion.

UC-MSC suspensions were prepared by various harvesting methods (Fig. 1). UC-MSCs were initially seeded into 35-mm tissue culture polystyrene (TCPS) dishes and cultured under the same conditions as those used for cell sheet fabrication. On Day 5, UC-MSCs were harvested as cell suspensions using various cell dissociation buffers that included 0.05% trypsin-EDTA (Gibco), TrypLE (Gibco), Accutase (Nacalai Tesque, Kyoto, Japan), and 1 mg/mL dispase II (Fujifilm Wako Pure Chemical, Osaka, Japan) or by cell scraping (Thermo Fisher Scientific, Waltham, MA, USA). UC-MSCs were treated with cell dissociation buffer for 5 min unless otherwise noted. To prepare the cell suspension with TR, 10-cm diameter temperature-responsive culture dishes (CellSeed, Tokyo, Japan) were used. On Day 5, UC-MSCs were treated with 0.05% trypsin-EDTA (Gibco) for 5 min and then re-seeded into 10-cm diameter temperature-responsive culture dishes. The cells were cultured for 2 days at 37 °C and then collected as a cell suspension by reducing the incubation temperature to 20 °C. Cells were plated in large temperature-responsive culture dishes (10-cm diameter) to prevent the formation of cell-cell connections, thus ensuring that they were collected as cell suspensions.

### 2.3 Preparation of UC-MSC sheets

UC-MSCs were seeded onto 35-mm diameter temperature-responsive culture dishes (CellSeed, Tokyo, Japan) at a density of 2 × 10^5^ cells/dish and cultured for 5 days until they reached confluence (Fig. 1D). The cell culture medium was replaced with a medium containing 20% FBS one day after seeding. On Day 5, UC-MSC cultures were harvested as intact monolayer sheets from temperature-responsive culture dishes within 60 min by reducing the incubation temperature to 20 °C.

### 2.4 Apoptosis detection of UC-MSCs

Apoptosis was assessed by Annexin V staining following the manufacturer's protocol using the Annexin V – fluorescein isothiocyanate (FITC) apoptosis detection kit (Nacalai Tesque). Annexin V-FITC and propidium iodide (PI) double staining were performed using an Annexin V-FITC apoptosis detection kit (Nacalai Tesque). Briefly, UC-MSCs were detached using different cell detachment methods (chemical or physical disruption) as described above. After washing the cell suspensions twice with phosphate buffered saline (PBS) (Gibco), they were resuspended in annexin V-binding buffer. The cells were then labeled with 10 μL of FITC-conjugated Annexin V and 10 μL of PI. Subsequently, the cells were incubated for 15 min in the dark at 37 °C, and 400 μL of binding buffer was added. Samples were immediately analyzed using a flow cytometer (BD Biosciences, Franklin Lakes, NJ, USA). The Annexin V-FITC (−)/PI (−) cell population was considered as live cells, whereas the Annexin V-FITC (+)/PI (−) cell populations were considered apoptotic cells, and Annexin V-FITC (+)/PI (+) cell populations were considered dead cells.

### 2.5 Lactate dehydrogenase assay

UC-MSCs were seeded into 35-mm diameter TCPS dishes and cultured. On Day 5, the cells were harvested either by reducing the incubation temperature to 20 °C or by chemical harvesting as described above. Subsequently, both cell sheets and cell suspensions were plated into 35-mm diameter TCPS dishes (Thermo Fisher Scientific) for 24 h, and the supernatant was collected. The lactate dehydrogenase (LDH) concentration was determined according to the manufacturer's protocol (Cytotoxicity LDH Assay Kit-WST, Dojindo, Kumamoto, Japan). Cells treated with 1% Triton X-100 were used as positive controls, and DMEM was used as a negative control. Relative cell viability was defined as the ratio of released LDH to total LDH in the positive control (1% Triton X-100 treated cells). All samples were assayed in triplicate.

### 2.6 Cell adhesion assay

The adhesion of single-cell suspensions was analyzed using a CCK-8 assay kit (Dojindo). Cell suspensions were prepared as described previously. After harvesting, the cell suspensions were plated into 24-well plates and incubated at 37 °C for 1, 3, and 6 h. Following incubation, the cells were washed with PBS (Gibco). CCK-8 solution (11-fold dilution in cell culture medium) was then added to each well, and this was followed by 1 h incubation at 37 °C. Optical absorbance was measured at 450 nm (570 nm was used as the reference wavelength) using a microplate reader (TECAN Infinite M1000, Männedorf, Switzerland). All samples were assayed in triplicate.

Cell sheet adhesion properties were investigated by microscopic observation of the edge of the cell sheet. FBS (1 mL) was pre-coated onto the TCPS plates for 1.5 h. UC-MSC sheets were prepared in temperature-responsive cell culture dishes. The prepared UC-MSC sheets were placed on FBS-coated TCPS plates, and the edges of the UC-MSC sheets were observed.

### 2.7 Detection of cytokine secretion by enzyme-linked immunosorbent assay

UC-MSCs were seeded into 35-mm diameter temperature-responsive culture dishes at a density of 2 × 10^5^ cells/dish and cultured for 5 days until they reached confluence. The cell culture medium was replaced with a medium containing 20% FBS at one day after seeding. On Day 5, UC-MSC sheets were harvested from temperature-responsive culture dishes by reducing the incubation temperature to 20 °C. To prepare the UC-MSC suspension, UC-MSCs were seeded onto 35-mm diameter culture dishes at a density of 2 × 10^5^ cells/dish and cultured for 5 days until they reached confluence. The UC-MSC suspension was recovered by trypsin treatment for 5 min. Subsequently, both UC-MSC sheets and cell suspensions containing the same number of cells were re-seeded onto the TCPS plates and incubated for 24 h. After incubation, the supernatants were collected and stored at −80 °C until enzyme-linked immunosorbent assay (ELISA) analysis. The cells were then detached and counted using the trypan blue exclusion assay. The levels of VEGF, HGF, IL-6, IL-10, and TGF-β1 in the supernatants from the cell sheets and cell suspensions were determined by ELISA (R&D Systems, Minneapolis, MN, USA) according to the manufacturer's recommendations. The cytokine levels were calculated using a standard curve constructed for each assay. DMEM containing 10% FBS (standard culture medium) was used as a negative control, and the cytokine levels in the negative control group were subtracted from each sample value.

### 2.8 Statistical analysis

All values are expressed as means ± SEM. Two-way analysis of variance followed by Tukey's post-hoc test was used to evaluate differences between more than two groups. Probabilities (e.g., p < 0.1, 0.05) were considered significant.

## 3 Results and discussion

### 3.1 Induced apoptosis of UC-MSCs during cell harvesting

**Fig. 2:**
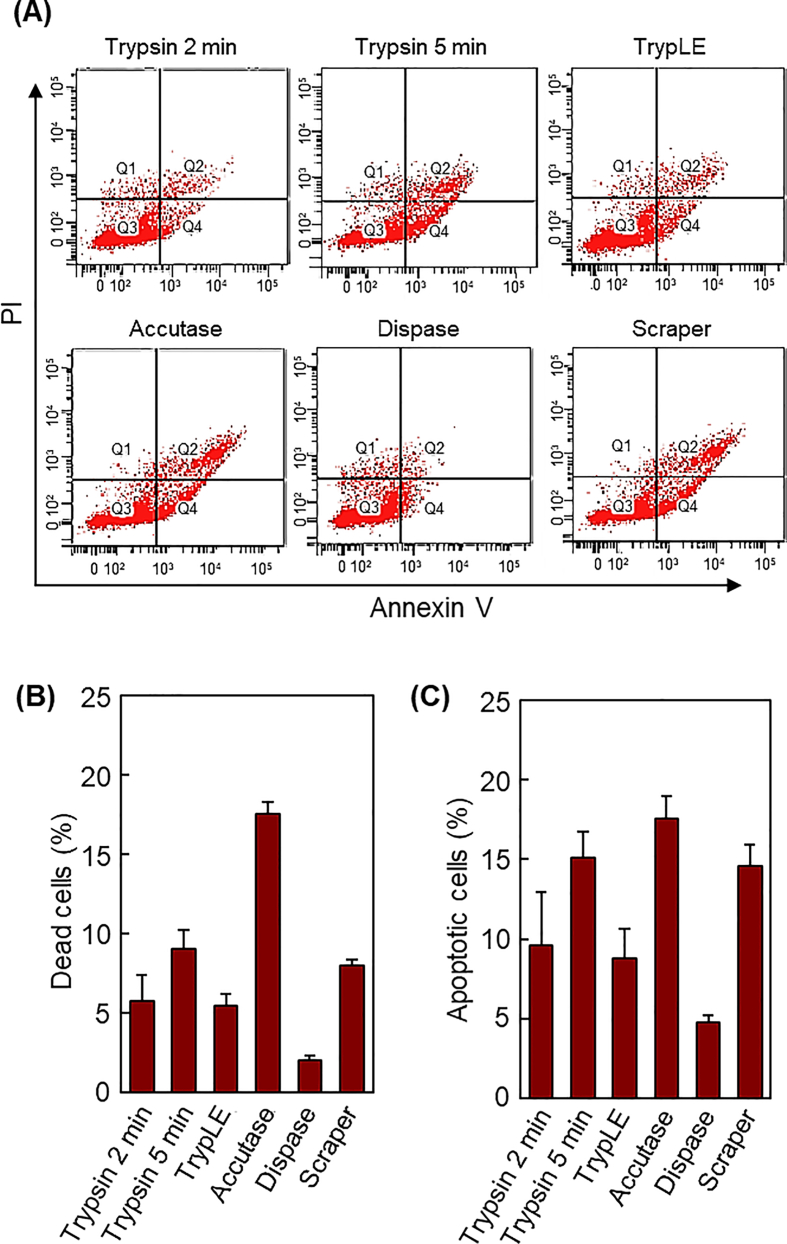
Apoptosis was assessed using the Annexin V assay. UC-MSCs were stained with Annexin V-FITC and Propidium Iodide (PI). (A) Flow cytometric analysis of recovered UC-MSCs, (B) percentage of dead cells, and (C) percentage of apoptotic cells (n = 3–5).

A previous report indicated that UC-MSCs harvested from culture dishes by enzymatic treatment with trypsin exhibited reduced cell activity and functions such as actin fiber formation and mechanosensor activity [70]. Various enzymes such as trypsin, TrypLE, accutase, and dispase are commonly used to harvest UC-MSCs from culture dishes. Additionally, a cell scraper was used to harvest the adherent cells (Fig. 1). Subsequently, UC-MSCs from the culture dishes were harvested and characterized using Annexin V binding assays (Fig. 2). A previous report indicated that UC-MSCs harvested from a PNIPAAm-modified temperature-responsive cell culture dish only slightly reduced the cell activity or function [70]. Therefore, UC-MSCs from temperature-responsive cell culture dishes were not investigated using Annexin V binding assays. Annexin V detects apoptosis according to cell surface layer changes that occur only when cells undergo apoptosis [71,72]. Normally, negatively charged phosphatidylserine (PS) exists inside the lipid bilayer; however, when apoptosis is induced, it is transferred to the outside of the lipid bilayer. Annexin V possesses a high affinity for PS in the presence of Ca^2+^ and thus binds only to cells in which PS is exposed on the cell surface by apoptosis. Furthermore, Propidium Iodide was used to stain the dead cells, thus enabling the detection of viable, early apoptotic, necrotic, and dead cells.

The Annexin V binding assay revealed that 0.05% trypsin treatment induced apoptosis and cell death in a time-dependent manner. Specifically, 5 min trypsin treatment resulted in relatively high proportions of dead and apoptotic cells compared to those in response to 2 min treatment (Fig. 2B and C). Among the cell harvesting methods investigated in this study, dispase exhibited the least cytotoxicity, with a relatively minor induction of apoptosis (4.8%) and cell death (2.0%) compared to that of the other methods. However, UC-MSCs treated with dispase for 5 min did not effectively detach from the culture dish, thus potentially leading to low cell survival rates in clinical studies where prolonged treatment is required to achieve a uniform cell suspension with dispase. TrypLE also exhibited low cytotoxicity (Fig. 2B and C). Adherent UC-MSCs were effectively harvested from the culture dish after 5 min of incubation with TrypLE, unlike the enzymatic harvesting with dispase. These results indicated that TrypLE could effectively harvest UC-MSCs from culture dishes with low cytotoxicity. Accutase exhibited a relatively high percentage of dead and apoptotic cells compared to that of the other types of enzymatic treatments. The detachment of UC-MSCs using Accutase was comparable to that using TrypLE and trypsin treatments. The results indicated that UC-MSC harvesting using Accutase reduced cellular activity compared to that of other enzymatic treatments. For UC-MSCs prepared by cell scraping, the ratio of dead to apoptotic cells was similar to that of cells treated with 0.05% trypsin for 5 min. These results indicated that the number of dead and apoptotic cells after enzymatic treatment varied with the type of enzyme used.

### 3.2 UC-MSC cell membrane injury during harvesting

**Fig. 3:**
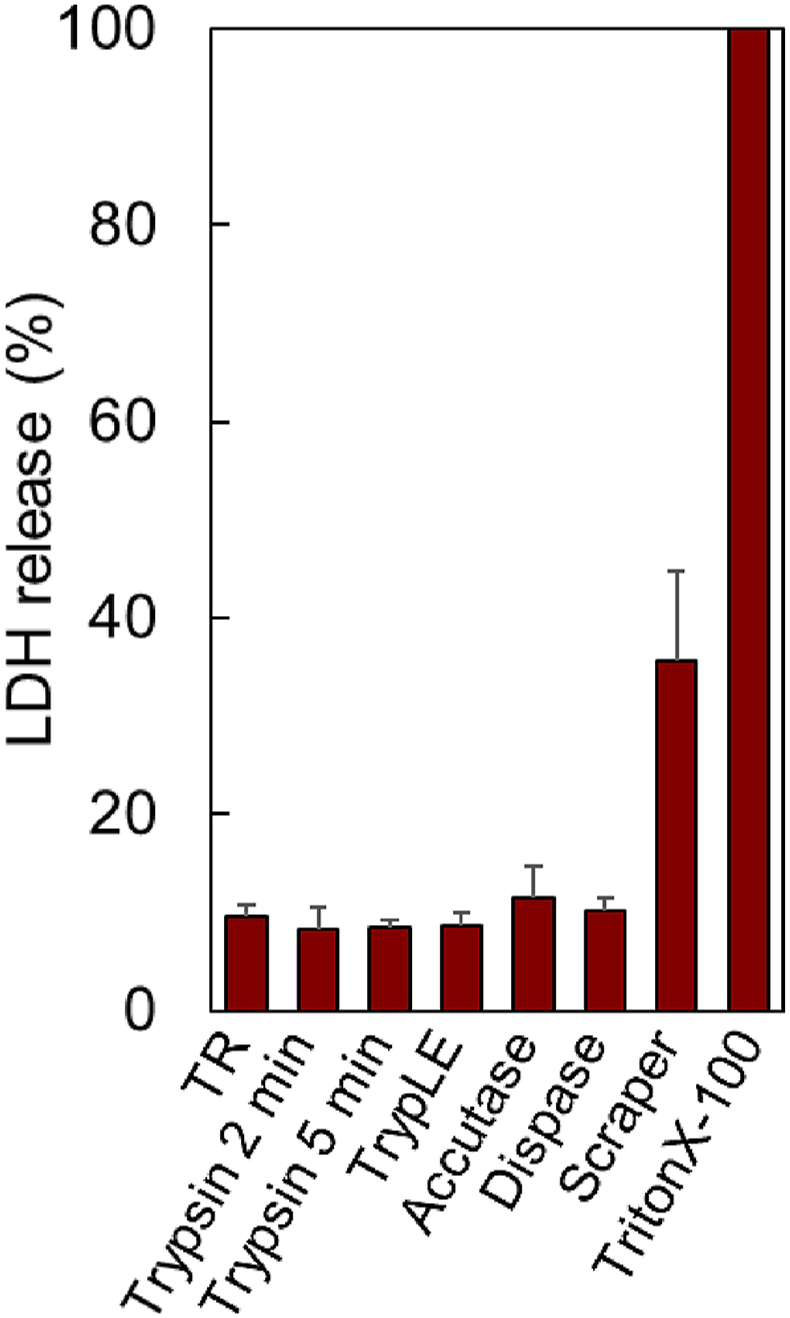
LDH assay of various types of UC-MSCs harvested from cell culture dishes. Cells were detached using various methods, re-seeded into TCPS, and cultured for 24 h. LDH release from the cell supernatant was measured. “TR” denotes UC-MSCs recovered from temperature-responsive cell culture dishes (n = 6–8).

Cell membrane injury in UC-MSCs prepared using various enzymes or cell scrapings was investigated using an LDH assay (Fig. 3). Additionally, cell membrane injury in UC-MSCs harvested from temperature-responsive cell culture dishes by changing the temperature was investigated.

LDH is an enzyme that resides in the cytoplasm and is released into the environment upon cell membrane injury. The LDH assay measures cell injury by quantifying the amount of LDH released into the culture medium [73,74].

The cell membranes were analyzed using LDH assays (Fig. 3). The LDH activity of the cells lysed with TritonX-100 was set to 100%. Although accutase exhibited a slightly higher LDH release than the other enzymatic digestions, significant differences in LDH release after all enzyme treatments were minimal. Additionally, UC-MSCs prepared using TR exhibited cytotoxicity similar to that of enzymatic treatments. These results indicated that cell membrane injury from all enzymatic treatments or TR was almost the same, as the LDH assay investigated LDH release after cell membrane injury. In contrast, cell scraping resulted in a higher LDH release than did enzymatic treatments and TR. These results indicate that the detachment of adherent cells using a cell scraper induces cell membrane injury.

### 3.3 UC-MSC re-adhesion behavior after cell harvesting

**Fig. 4:**
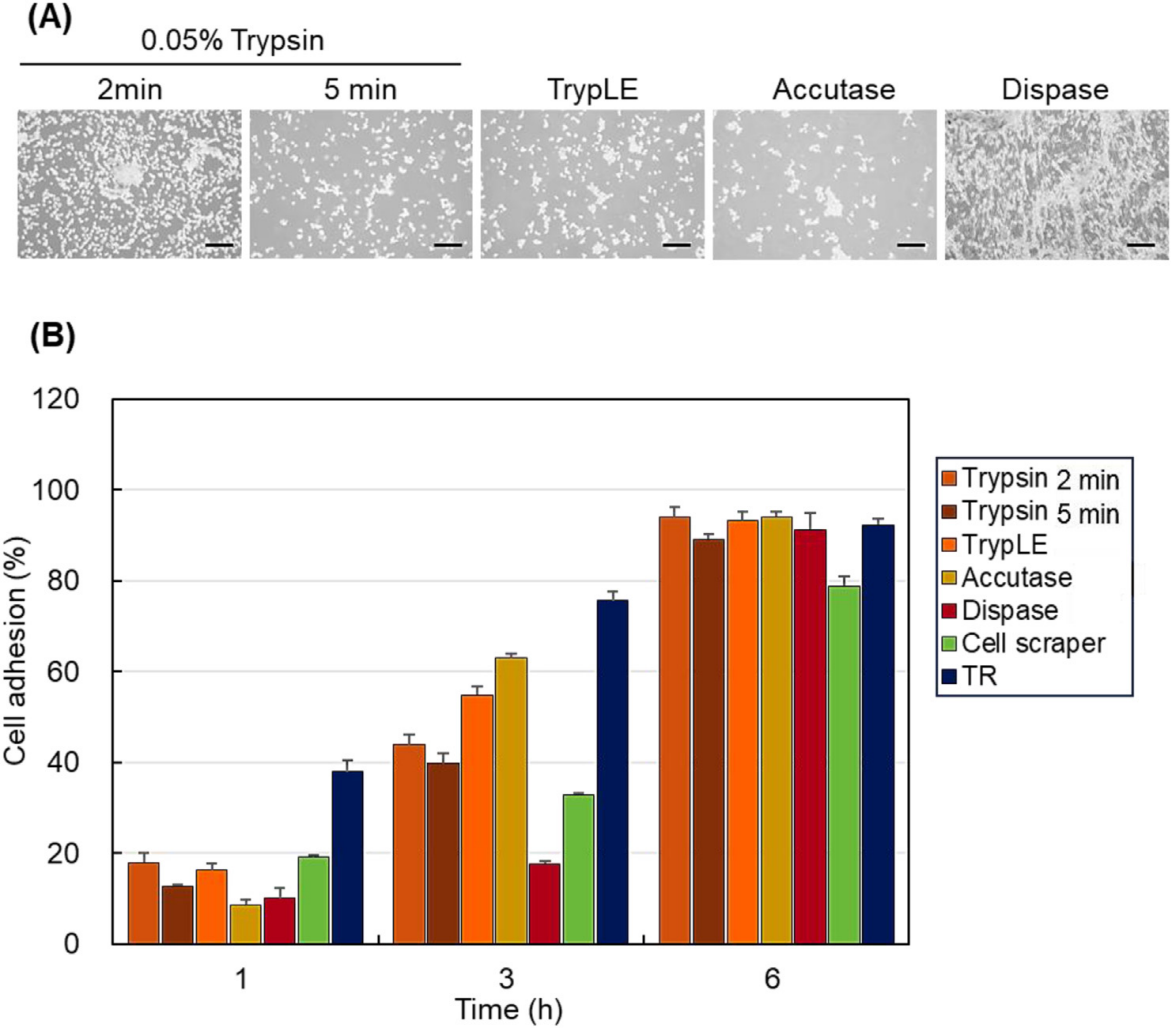
The re-adhesion of UC-MSCs was assessed using CCK-8 assays. (A) UC-MSCs were harvested with various enzymatic treatments. The microscopic images of re-seeded UC-MSCs into 24-well cell culture plates after 15 min incubation. Scale bar: 100 μm. (B) The cell adhesion ratios of UC-MSC suspensions prepared using various enzymes. “TR” denotes the UC-MSC recovered from temperature-responsive cell culture dishes (n = 3).

To examine the effect of different cell detachment methods on UC-MSC re-adhesion, the number of adherent cells on the culture plates after re-adhesion was determined using CCK-8 assays (Fig. 4). UC-MSCs were treated with various proteolytic enzymes (0.05% trypsin, accutase, dispase, and TrypLE), cell scraping, or TR and then reseeded into 24-well cell culture plates (Fig. 4A). The number of adherent cells after 1, 3, and 6 h of incubation following reseeding was analyzed using the CCK-8 assay (Fig. 4B).

The cell adhesion ratio increased with incubation time after re-seeding, and most of the re-seeded UC-MSCs were attached after 6 h (Fig. 4B). At 1 h, TR exhibited relatively high cell adhesion compared to that of trypsin-treated cells and after cell scraping. This was due to the observation that the UC-MSC harvested after TR maintained their extracellular matrix. Conversely, other enzymatic treatments and cell scraping exhibited low cell adhesion, likely due to the destruction of the extracellular matrix of UC-MSCs by these methods. At 3 h, TR exhibited relatively high adhesion compared to that of the enzymatic treatments and cell scraping. Among the enzymatic treatments, dispase treatment resulted in a remarkably low adhesion ratio. These results indicate that dispase induces extensive destruction of the extracellular matrix and cell adhesion proteins compared to that of other enzymatic treatments. Moreover, cell scraping resulted in a remarkably low adhesion ratio, likely due to the physical destruction of the extracellular matrix and cell adhesion proteins. At 6 h, the cell adhesion ratios for all the detachment methods were similar. These results suggested that the cell adhesion capacity of UC-MSCs recovered after 6 h of incubation. The investigation of UC-MSC re-adhesion revealed that treatment with dispase or cell scraping reduced the cell re-adhesion function until 3 h of incubation; however, the adhesive capacity was almost recovered after 6 h of incubation.

**Fig. 5:**
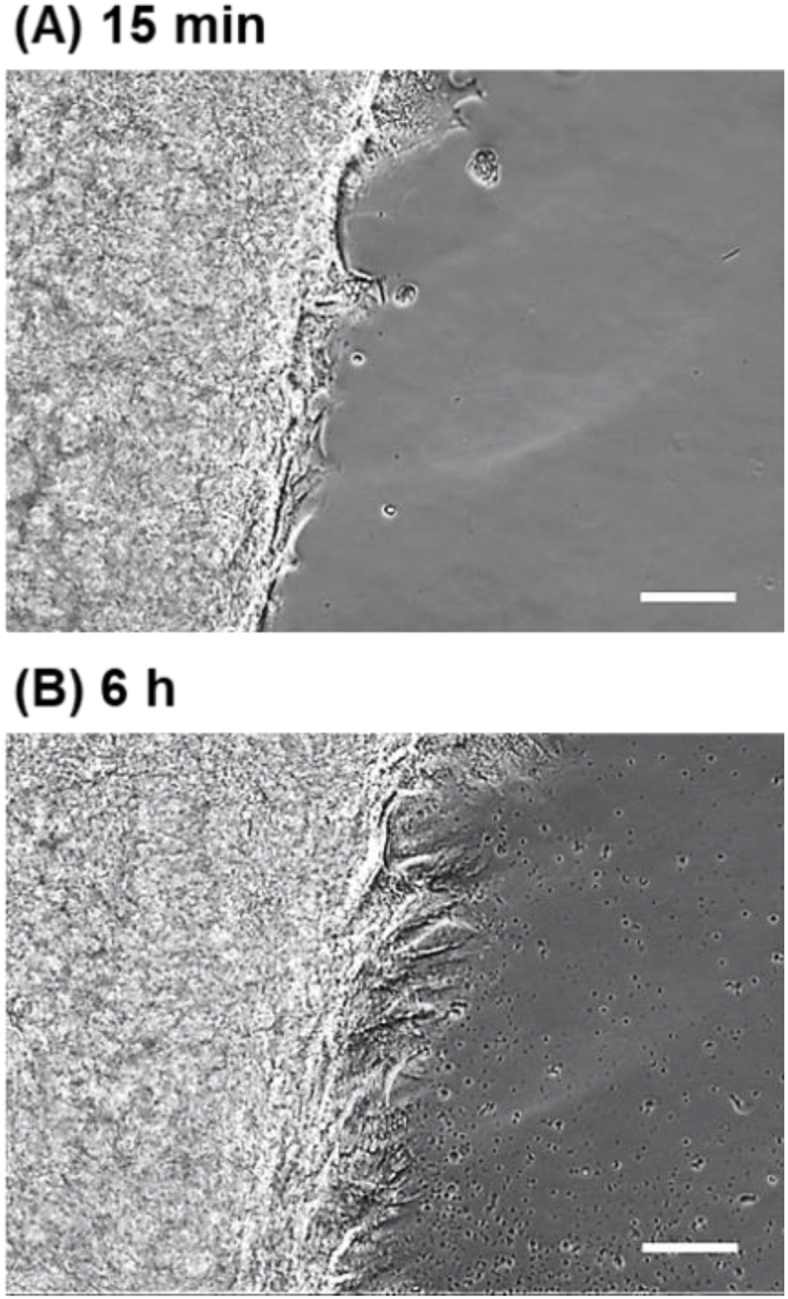
Re-adhesion behavior of UC-MSC sheets on TCPS. (A) Adhered UC-MSC sheet on TCPS after re-adhesion at 15 min. (B) Adhered UC-MSC sheet on TCPS after re-adhesion at 6 h. Scale bar: 100 μm.

To confirm the adhesive and migratory properties of UC-MSC sheets, their adhesion behavior on FBS-coated TCPS plates was observed (Fig. 5). Typically, cells adopt a spindle shape in an adhesive state in culture dishes. After re-adhesion of the UC-MSC sheet for 15 min, the cells in the sheet exhibited a spindle shape (Fig. 5A). The results indicate that the cells adhered to the TCPS even after 15 min. To confirm the adhesion of the UC-MSC sheet to TCPS, the adherent UC-MSC sheet was gently rinsed with PBS. The adhered UC-MSCs did not flow into the PBS and maintained their adhesion to TCPS. After 6 h of incubation, the cell sheets migrated (Fig. 5B). These results indicated that UC-MSCs maintained high activity even after detachment and re-adhesion.

### 3.4 Cytokine secretion by UC-MSC sheets

**Fig. 6:**
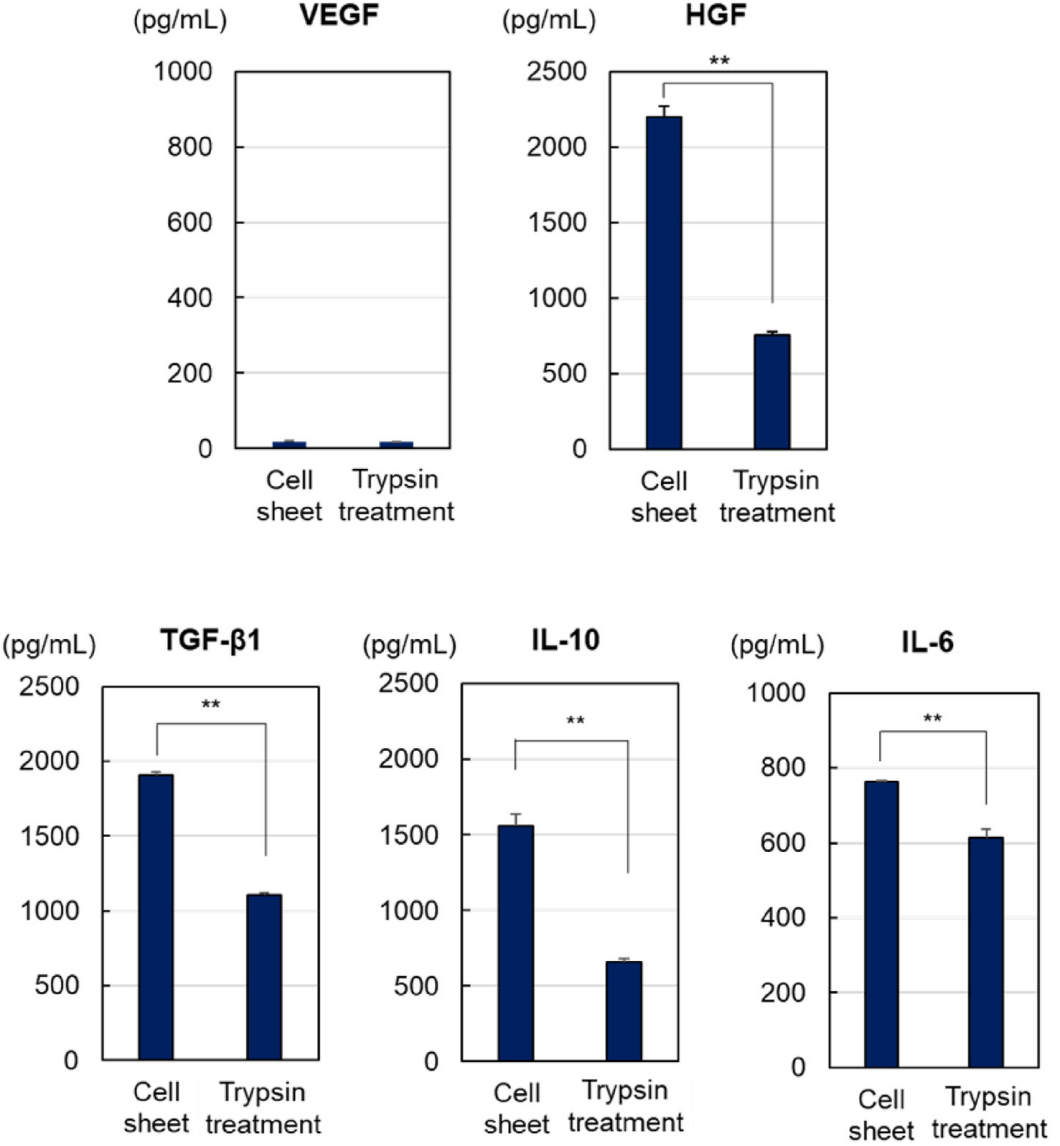
Cytokine secretion by the adhered UC-MSC sheets. The UC-MSC sheet was detached from the temperature-responsive cell culture dish and adhered to the TCPS. Cytokine expression in the cell culture medium was determined using ELISA (n = 3, ∗p < 0.05; ∗∗p < 0.01).

To investigate differences in secretion between UC-MSC sheets and cell suspensions, the levels of secreted cytokines (VEGF, HGF, TGF-β1, IL-10, and IL-6) in the culture medium were measured by ELISA after incubation for 24 h (Fig. 6). The amount of VEGF in the culture medium was low for both UC-MSC sheets and cell suspensions, likely due to VEGF secretion by UC-MSCs being suppressed *in vitro*. UC-MSC sheets exhibited remarkably high HGF, TGF-β1, and IL-10 secretion levels compared to those of the UC-MSC suspension. Additionally, relatively high secretion of IL-6 was observed in UC-MSC sheets compared to that of the UC-MSC suspensions. This was attributed to the UC-MSC sheet maintaining its activity compared to that of the UC-MSC suspension. UC-MSC sheets were harvested using TR without reducing cellular activity. Furthermore, the cell-cell connections of each cell were maintained in the UC-MSC sheets, ultimately leading to high functionality of each cell in the cell sheet. Thus, cytokine production was maintained after re-adhesion of the cell sheets. In contrast, the UC-MSC suspension was harvested from the culture via trypsin digestion, and this reduced cell activity. The harvested and reseeded UC-MSC suspensions consume energy for the reconstruction of cellular structures for cell adhesion, ultimately leading to relatively low cytokine secretion. An investigation of cytokine secretion revealed that the UC-MSC sheet secreted more cytokines than did the trypsin-treated cell suspension.

These results indicated that enzymatic treatment and cell scraping induced cell apoptosis, damaged the cell membrane, and reduced cell adhesion properties compared to that in response to temperature-modulated cell harvesting. Furthermore, the UC-MSC sheets secreted large amounts of cytokines compared to that of the UC-MSC suspension *in vitro*. Thus, cell harvesting methods influence the function and activity of UC-MSCs.

## 4 Conclusions

We investigated the properties and functions of UC-MSCs harvested using various enzymatic treatments, scraping with a cell scraper, and the TR method in temperature-responsive cell culture dishes. Apoptosis during cell harvesting from culture dishes was investigated using annexin V. Accutase-induced apoptosis in UC-MSCs was more effective than were other cell harvesting methods. The LDH assay for investigating cell membrane injury revealed that physical scraping using a cell scraper induced a relatively high LDH release due to cell membrane damage compared to that in response to other types of enzyme treatments. The adhesion properties of UC-MSCs were investigated using the CCK-8 assay. After an initial incubation for 3 h, the UC-MSC suspension harvested using dispase exhibited relatively low cell adhesion compared to that of the other enzymatic treatments. UC-MSC sheet re-adhesion was observed even after 15 min, and cell migration was observed after 6 h. UC-MSC sheets exhibited high levels of cytokine expression, including HGF, TGF-β1, IL-10, and IL-6, compared to that of the UC-MSC suspension.

## Author contributions

MN and KN conceived the study. KN supervised the study. MN and KN designed experiments. MN obtained experimental data. MN and KN wrote the main manuscript text and reviewed the manuscript.

## Funding

This study was partially supported by Grants-in-Aid for Scientific Research (grant numbers 19H02447, 21KK0199, 22K19899, 20H05233, 22H04560, and 24K01181) from the Japan Society for the Promotion of Science, the research grant of Precise Measurement Technology Promotion Foundation (PMTP-F), the research grant of Iketani Science Technology Foundation, and the research grant of CASIO Science Promotion Foundation.

## Declaration of competing interest

The authors declare that they have no known competing financial interests or personal relationships that could have appeared to influence the work reported in this paper.
